# IL-17 Facilitates VCAM-1 Production and Monocyte Adhesion in Osteoarthritis Synovial Fibroblasts by Suppressing miR-5701 Synthesis

**DOI:** 10.3390/ijms23126804

**Published:** 2022-06-18

**Authors:** Tsung-Ju Wu, Sunny Li-Yun Chang, Chih-Yang Lin, Chao-Yang Lai, Xiu-Yuan He, Chun-Hao Tsai, Chih-Yuan Ko, Yi-Chin Fong, Chen-Ming Su, Chih-Hsin Tang

**Affiliations:** 1 Department of Physical Medicine and Rehabilitation, Changhua Christian Hospital, Changhua 500209, Taiwan; 134330@cch.org.tw; 2 Graduate Institute of Biomedical Sciences, China Medical University, Taichung 404333, Taiwan; sunnylyc@gmail.com (S.L.-Y.C.); hchp99812@gmail.com (X.-Y.H.); 3 Translational Medicine Center, Shin-Kong Wu Ho-Su Memorial Hospital, Taipei 111045, Taiwan; T016406@ms.skh.org.tw; 4 Department of Medical Laboratory Science and Biotechnology, Asia University, Taichung 413305, Taiwan; chaoyang@asia.edu.tw; 5 Department of Sports Medicine, College of Health Care, China Medical University, Taichung 406040, Taiwan; ritsai8615@gmail.com (C.-H.T.); yichin.fong@gmail.com (Y.-C.F.); 6 Department of Orthopedic Surgery, China Medical University Hospital, Taichung 404327, Taiwan; d14333@mail.cmuh.org.tw; 7 Department of Orthopaedic Surgery, China Medical University Beigang Hospital, Yunlin 651012, Taiwan; 8 Department of Pharmacology, School of Medicine, China Medical University, Taichung 406040, Taiwan; 9 Chinese Medicine Research Center, China Medical University, Taichung 406040, Taiwan

**Keywords:** IL-17, osteoarthritis, monocytes, VCAM-1, adhesion

## Abstract

Osteoarthritis (OA) is characterized by the infiltration and adhesion of monocytes into the inflamed joint synovium. Interleukin (IL)-17 is a critical inflammatory mediator that participates in the progression of OA, although the mechanisms linking IL-17 and monocyte infiltration are not well understood. Our analysis of synovial tissue samples retrieved from the Gene Expression Omnibus (GEO) dataset exhibited higher monocyte marker (CD11b) and vascular cell adhesion molecule 1 (VCAM-1) levels in OA samples than in normal, healthy samples. The stimulation of human OA synovial fibroblasts (OASFs) with IL-17 increased VCAM-1 production and subsequently enhanced monocyte adhesion. IL-17 affected VCAM-1-dependent monocyte adhesion by reducing miR-5701 expression through the protein kinase C (PKC)-α and c-Jun N-terminal kinase (JNK) signaling cascades. Our findings improve our understanding about the effect of IL-17 on OA progression and, in particular, VCAM-1 production and monocyte adhesion, which may help with the design of more effective OA treatments.

## 1. Introduction

Osteoarthritis (OA) is a joint disorder that is accompanied by the migration and invasion of monocytes into the synovial membrane, leading to synovial inflammation, cartilage degradation, and bone breakdown [1,2], evoking pain and adversely affecting the patient’s quality of life. As the disease progresses, critical steps in the joint microenvironment regarding the synthesis of proinflammatory factors and chondrolytic mediators enhance the breakdown of cartilage and loss of bone [3,4]. Numerous OA synovial fibroblasts (OASFs) in the joint microenvironment control the development of OA by synthesizing proinflammatory factors and catabolic mediators [3,5,6], indicating that remedying the state of the synovium is appropriate for OA treatment [7,8].

Macrophage and monocyte infiltration into the joint microenvironment and their adhesion to the synovial membrane is an important mediator of OA development [9]. Several adhesion molecules regulate the infiltration and migration of macrophages and monocytes during OA development, including vascular cell adhesion molecule 1 (VCAM-1) [9,10]. Higher VCAM-1 expression is documented in human OA synovium than in normal synovium [10]. The inhibition of VCAM-1 levels in OA synovium reportedly lowers the inflammatory response during OA progression [11,12].

Accumulating evidence indicates that the interleukin (IL)-17 family of cytokines regulates the pathogenesis of OA [13]. A higher expression of IL-17 in OA serum and synovial fluid is reflected by radiographic OA severity scores [13,14]. *IL-17* gene polymorphisms have been linked to the development of OA in several populations [15,16], while injections of IL-17 into the rabbit knee joint induces OA [17]. Moreover, IL-17 enhances osteoclastogenesis, and bone erosion plays an important role in arthritic diseases [18]. Thus, it is worth targeting IL-17 as a novel therapeutic for managing OA disease.

MicroRNAs (miRNAs, single-stranded noncoding RNAs) regulate the production of target genes at the post-transcriptional stage [19,20]. Several miRNAs mediate the development of OA by negatively or positively regulating synovial cell inflammation, differentiation, angiogenesis, and survival [21,22]. However, it is not clear as to whether IL-17 regulates miRNA-mediated VCAM-1 synthesis and the adhesion of monocytes during OA progression. Here, we found higher levels of the monocyte marker CD11b and VCAM-1 expression in OA synovial tissue than in normal, healthy tissue. Our results also indicate that IL-17 facilitates VCAM-1 production and promotes monocyte adhesion in human OASFs by decreasing miR-5701 expression in the protein kinase C (PKC)-α and c-Jun N-terminal kinase (JNK) signaling cascades, indicating that IL-17 may be worth targeting when treating OA.

## 2. Results

### 2.1. IL-17 Promotes Monocyte Adhesion in Human OASFs by Enhancing VCAM-1 Production

**Figure 1:**
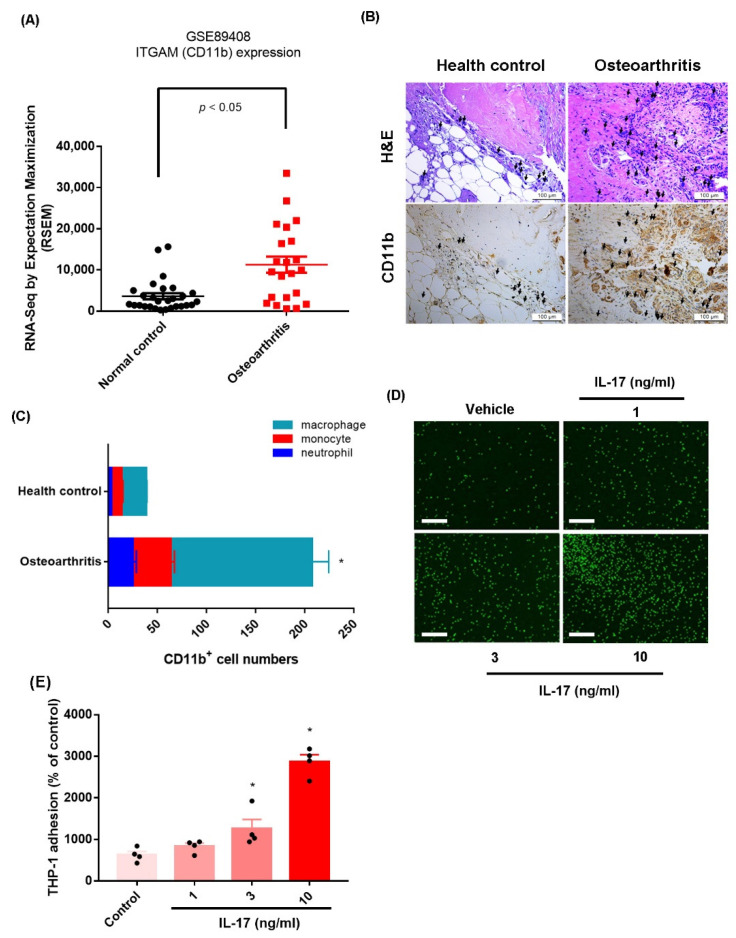
**IL-17 promotes monocyte adhesion in OASFs.** (**A**) Levels of CD11b were investigated in OA and healthy control synovial tissue collected from the GEO database. (**B**,**C**) IHC staining for CD11b^+^ (neutrophil, monocyte, and macrophage) in synovium samples from OA patients and healthy individuals. (**D**,**E**) OASFs were stimulated with vehicle or IL-17 (1–10 ng/mL) for 24 h. Monocytes (THP-1 cells) were then applied to the OASFs. Adherent THP-1 cells were photographed and quantified under fluorescence microscopy. Size bar = 320 μm. * *p* < 0.05 compared with the control group.

The infiltration of macrophages and monocytes and their adhesion to synovial membrane, where they promote a proinflammatory response, is a critical step in OA development [23]. Since CD11b is a major monocyte marker for OA disease, we examined the expression of CD11b in raw data from OA (*n* = 22) and healthy synovial tissue samples (*n* = 28) downloaded from the Gene Expression Omnibus (GEO) dataset (accession code: GSE89408). The analyses revealed significantly higher levels of the monocyte marker CD11b in OA synovial samples than in normal controls (Figure 1A), which was also the case in our histopathologic analysis of CD11b expression in our samples of human OA synovial tissue (*n* = 4) and healthy control samples (*n* = 4) (Figure 1B,C, Supplementary Figure S1A). OASFs were stimulated with IL-17 for 2 h, and then the culture was changed to a serum-free medium for the next 24 h (to exclude the direct effect of IL-17 on monocytes), and monocytes (THP-1 cells) were added to a monolayer of OASFs for 1 h. Monocyte adhesion analysis revealed that IL-17 treatment dose-dependently enhanced monocyte adhesion in OASFs (Figure 1D,E, Supplementary Figure S1B). Treatment of monocytes with IL-17 for 1 h did not increase their adherence to OASFs (Supplementary Figure S2). Stimulation of OASFs with TNF-α also promoted monocyte adherence (Supplementary Figure S3). Thus, IL-17 promotes monocyte adhesion in human OASFs; the other pathways, such as TNF-α, also have similar effects.

**Figure 2:**
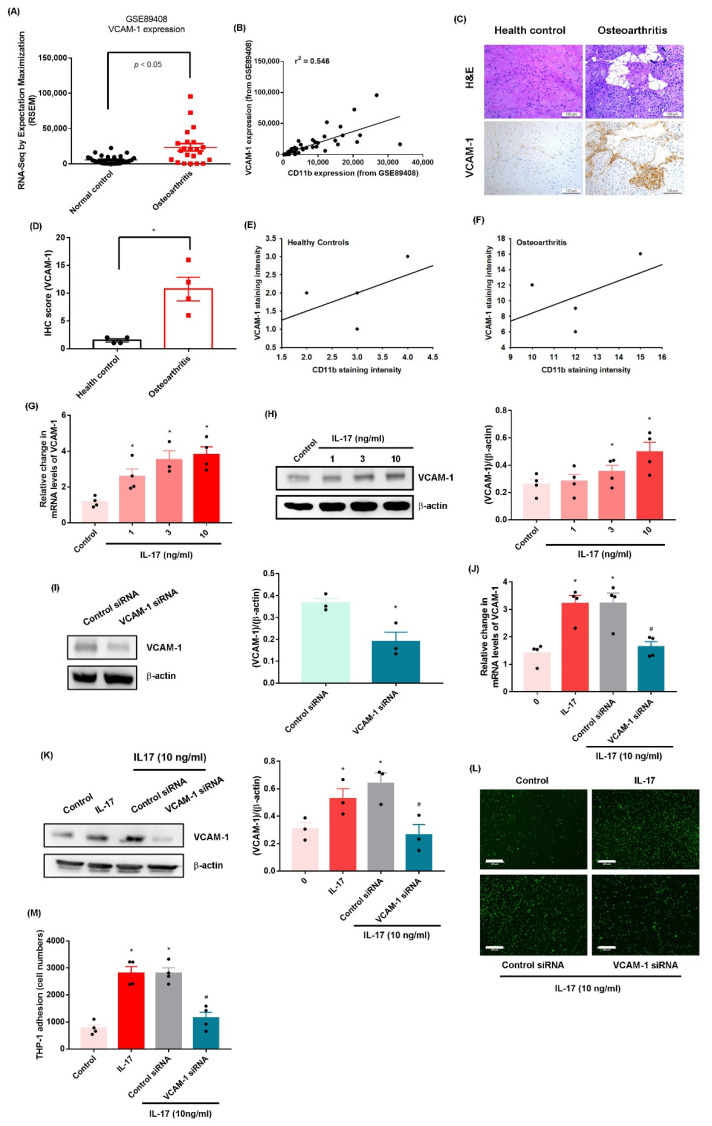
**IL-17 promotes monocyte adhesion in OASFs through upregulating VCAM-1 production.** (**A**) Levels of VCAM-1 were investigated in synovial tissue collected from the GEO database containing OA patients and healthy controls. (**B**) Spearman’s rank correlation coefficient testing identified significant, positive correlations between VCAM-1 and CD11b. (**C**–**F**) IHC staining for VCAM-1 in synovium samples from OA patients and healthy individuals. (**G**,**H**) OASFs were stimulated with IL-17 for 24 h, and VCAM-1 synthesis was performed by qPCR and Western blot. (**I**–**M**) OASFs were transfected with a VCAM-1 siRNA for 24 h and then treated with or without IL-17 (10 ng/mL). VCAM-1 expression was examined by Western blot and qPCR. Adherent THP-1 cells were photographed and quantified under fluorescence microscopy. Size bar = 320 μm. * *p* < 0.05 compared with the control group; # *p* < 0.05 compared with the IL-17-stimulated group.

VCAM-1-regulated adhesion of macrophages and monocytes to the synovial membrane is crucial for OA [24]. Data from the GEO database and our clinical samples displayed higher expression of VCAM-1 in OA synovial tissue than in healthy tissue (Figure 2A–D, Supplementary Figure S4A), and we observed a significant positive correlation between CD11b and VCAM-1 levels (Figure 2E,F). Treatment of OASFs with IL-17 promoted the transcription of VCAM-1 mRNA and the translation of VCAM-1 protein (Figure 2G,H). Transfection of OASFs with VCAM-1 siRNA without IL-17 treatment inhibited VCAM-1 expression (Figure 2I). In addition, transfection of OASFs with VCAM-1 siRNA and IL-17 treatment abolished IL-17-induced promotion of VCAM-1 expression and monocyte adhesion (Figure 2J–M, Supplementary Figure S4B). VCAM-1 siRNA completely inhibited IL-17-induced promotion of VCAM-1 expression. Thus, IL-17 facilitates the adhesion of monocytes to human OASFs by facilitating VCAM-1 synthesis.

### 2.2. IL-17 Promotes VCAM-1-Dependent Monocyte Adhesion in OASFs via the PKC-α and JNK Signaling Pathways

**Figure 3:**
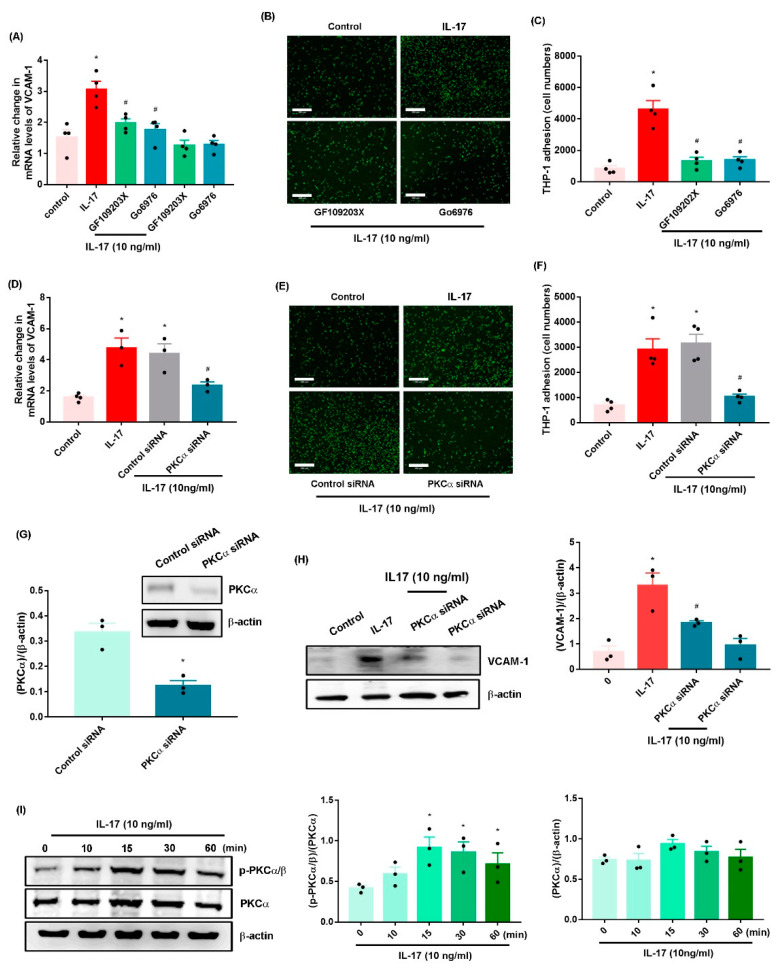
**The PKC-α pathway mediates IL-17-induced promotion of VCAM-1 synthesis and monocyte adhesion in OASFs.** (**A**,**D**,**H**) OASFs were stimulated with PKC inhibitors (GF109203X, 10 nM and Gö6976, 10 nM) for 30 min or transfected with a PKC-α siRNA for 24 h and then treated with or without IL-17 (10 ng/mL) for 24 h. Quantification of VCAM-1 expression was performed by qPCR and Western blot. (**B**,**C**,**E**,**F**) Adherent THP-1 cells were photographed and quantified under fluorescence microscopy. (**G**) PKC-α protein levels were measured by Western blot. (**I**) OASFs were stimulated with IL-17 for the indicated time intervals. PKC-α phosphorylation was performed by Western blot. Size bar = 320 μm. * *p* < 0.05 compared with the control group; # *p* < 0.05 compared with the IL-17-stimulated group.

PKC activation is an important event in OA development [12,25]. Treatment of OASFs with the pan PKC inhibitor (GF109203X) and PKC-α/β inhibitor (Gö6976) or PKC-α siRNA antagonized IL-17-induced increases in VCAM-1 synthesis and monocyte adhesion (Figure 3A–F, Supplementary Figure S5A,B). The PKC inhibitors (GF109203X and Gö6976) did not affect the basal levels of VCAM-1 mRNA expression (Figure 3A). Transfection of OASFs with PKC-α siRNA inhibited PKC-α expression (Figure 3G); the knockdown of PKC-α produced similar effects (Figure 3H). In addition, Western blot analysis found that IL-17 facilitated PKC-α phosphorylation (Figure 3I).

**Figure 4:**
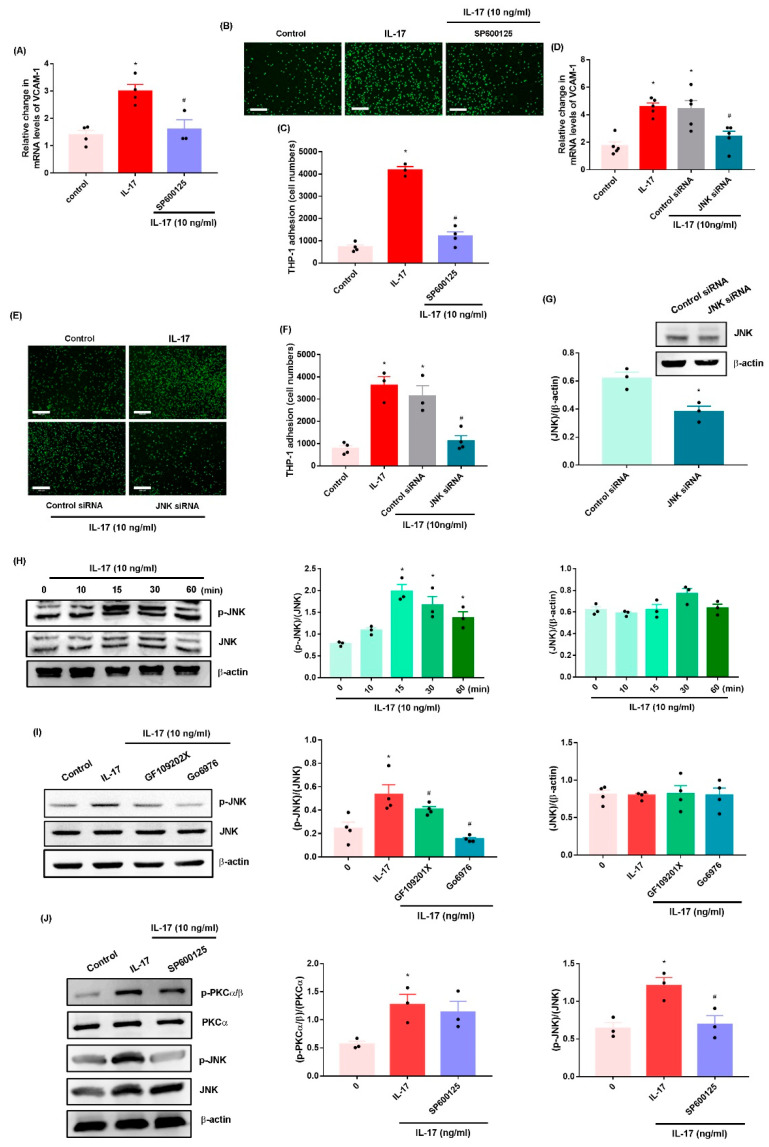
**The JNK pathway mediates IL-17-induced promotion of VCAM-1 synthesis and monocyte adhesion in OASFs.** (**A**,**D**) OASFs were stimulated with a JNK inhibitor (SP600125, 10 nM) for 30 min or transfected with a JNK siRNA for 24 h and then treated with IL-17 (10 ng/mL) for 24 h. VCAM-1 levels were quantified by qPCR. (**B**,**C**,**E**,**F**) Adherent THP-1 cells were photographed and quantified under fluorescence microscopy. (**G**) JNK protein levels were measured by Western blot. (**H**–**J**) OASFs were stimulated with IL-17 for the indicated time intervals or pretreated with PKC or JNK inhibitors and then incubated with IL-17 (10 ng/mL). JNK or PKC phosphorylation was performed by Western blot. Size bar = 320 μm. * *p* < 0.05 compared with the control group; # *p* < 0.05 compared with the IL-17-stimulated group.

JNK is a downstream molecule of PKC in VCAM-1-mediated cell adhesion [12]. Both the JNK inhibitor (SP600125) and JNK siRNA reversed IL-17-induced promotion of VCAM-1 synthesis and monocyte adhesion (Figure 4A–F, Supplementary Figure S6A,B). Transfection of OASFs with JNK siRNA inhibited JNK expression (Figure 4G). IL-17 also enhanced the phosphorylation of JNK (Figure 4H), which was diminished by pretreatment with the PKC inhibitors (Figure 4I). In contrast, the JNK inhibitor inhibited IL-17-promoted JNK but not PKC-α phosphorylation (Figure 4J), suggesting that PKC-α-dependent JNK activation mediates IL-17-induced enhancement of VCAM-1 synthesis and monocyte adhesion in OASFs.

### 2.3. IL-17 Enhances VCAM-1 Synthesis and Monocyte Adhesion by Suppressing miR-5701 Expression

**Figure 5:**
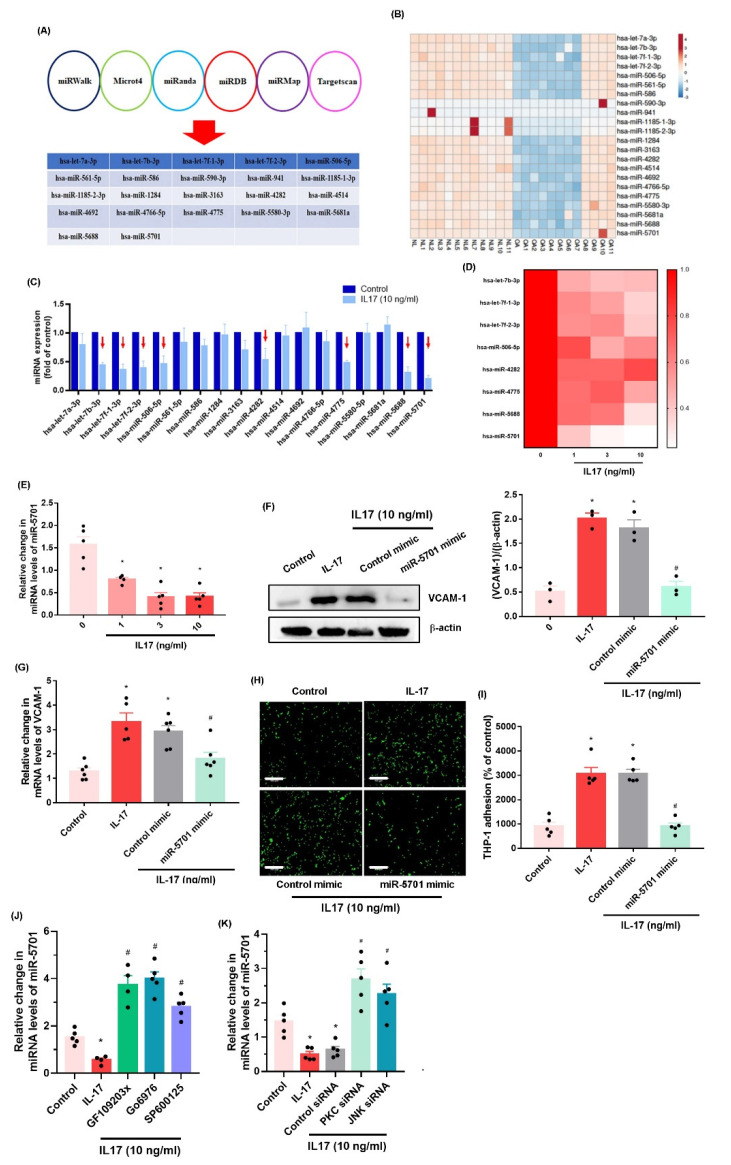
**IL-17 enhances VCAM-1 production by inhibiting miR-5701 expression.** (**A**) Six software databases were examined to predict which miRNAs interfere with VCAM-1 transcription. (**B**) Levels of miRNAs were investigated in OA and normal, healthy synovial tissue collected from the GEO database. (**C**–**E**) OASFs were treated with IL-17 (10 ng/mL). miRNA expression was examined by qPCR. (**F**,**G**) OASFs were transfected with miR-5701 mimic and then treated with IL-17 (10 ng/mL). VCAM-1 expression was examined by Western blot and qPCR. (**H**,**I**) Adherent THP-1 cells were photographed and quantified under fluorescence microscopy. (**J**,**K**) OASFs were stimulated with PKC and JNK inhibitors for 30 min or the respective siRNAs for 24 h and then incubated with IL-17 (10 ng/mL). miR-5701 expression was examined by qPCR. Size bar = 320 μm. * *p* < 0.05 compared with the control group; # *p* < 0.05 compared with the IL-17-stimulated group.

Numerous miRNAs are found at different levels of expression in normal and OA synovial tissue and regulate OA progression [26,27]. Analyses of six open-source software programs predicted that 21 miRNAs interfere with VCAM-1 mRNA transcription (Figure 5A). Our analysis of the GEO database found that among these miRNAs, eighteen (including miR-5701) exhibited lower levels of expression in OA patients than in normal, healthy individuals (Figure 5B). When OASFs were treated with IL-17, the expression of miR-5701 was suppressed by a markedly greater extent compared with the other miRNAs (Figure 5C). In addition, IL-17 concentration-dependently abolished miR-5701 synthesis (Figure 5D,E). Transfecting OASFs with miR-5701 mimic lowered VCAM-1 synthesis and monocyte adhesion (Figure 5F–I, Supplementary Figure S7). Treating OASFs with inhibitors and siRNAs of PKC-α and JNK antagonized IL-17-induced inhibition of miR-5701 synthesis (Figure 5J,K), indicating that IL-17 suppresses miR-5701 synthesis through the PKC-α and JNK signaling pathways.

## 3. Discussion

OA causes much physical disability [1]. Much remains unknown about the pathogenesis of OA; however, synovial inflammation is well-recognized [28], so treatment targeting the synovium may inhibit disease progression [7,29]. Elevated levels of macrophage and monocyte expression are found in the OA joint [30]. In this study, an analysis of the GEO database and our clinical results found higher levels of the monocyte marker CD11b in synovial tissue from OA patients than in tissue from normal, healthy individuals. IL-17 appears to aggravate the symptoms of OA [13]. Higher IL-17 levels have been reported in the serum and synovial fluid of OA patients than in healthy controls [13,14]. In this study, we demonstrated that IL-17 enhanced monocyte adhesion in human OASFs by upregulating VCAM-1 production. Moreover, the suppression of miR-5701 expression via PKC-α and JNK signaling regulated the effects of IL-17. The anti-IL17 monoclonal antibody secukinumab is approved for the treatment of psoriasis [31]. Whether anti-IL-17 agents are appropriate in OA treatment needs to be clarified.

VCAM-1-dependent mononuclear cell infiltration and adhesion in synovial tissue influences arthritic progression [24,32]. In this study, the GEO database records and our clinical samples displayed higher VCAM-1 expression in synovial tissue from OA patients compared with that from normal, healthy controls. A positive correlation between CD11b and VCAM-1 levels indicates that these cytokines contribute to OA disease progression. Here, we found that VCAM-1 is a response mediator in IL-17 stimulation, promoting monocyte adhesion. This effect was antagonized when OASFs were transfected with VCAM-1 siRNA, which suggests that IL-17 facilitates VCAM-1-induced monocyte adhesion in human OASFs.

PKC activation is crucial for regulating different cellular events [33], such as the promotion of cell motility and adhesion [12,34]. Our data found that IL-17 enhances PKC-α phosphorylation, while the PKC inhibitor or siRNA diminished IL-17-facilitated promotion of VCAM-1 synthesis and monocyte adhesion in OASFs. JNK is essential for regulating the inflammatory process during OA disease [35,36]. Our data showed that a JNK inhibitor or siRNA antagonized IL-17-facilitated VCAM-1-dependent monocyte adhesion. Our findings also reveal that IL-17 enhances JNK phosphorylation, which was reversed by the PKC inhibitor, indicating that PKC-α-dependent JNK activation regulates IL-17-induced synthesis of VCAM-1 and monocyte adhesion in human OASFs.

miRNAs are critical post-transcriptional mediators of gene production and are found in several diseases, including OA [37,38]. It has been proposed that pharmacotherapy capable of controlling miRNA levels would inhibit OA inflammatory progression and thus be an appropriate therapeutic approach for this disease [37,39]. Here, our analysis of six miRNA software databases predicted that miR-5701 interferes with VCAM-1 transcription. This was supported by the study data showing lower miR-5701 expression in OA synovial tissue than in normal, healthy tissue. We found that IL-17 treatment inhibits miR-5701 expression, and treatment of OASFs with miR-5701 mimic antagonizes IL-17-induced promotion of VCAM-1 synthesis and monocyte adhesion. PKC-α and JNK inhibitors, as well as their respective siRNAs, antagonized IL-17-enhanced inhibition of miR-5701 synthesis, suggesting that IL-17 promotes VCAM-1 synthesis and monocyte adhesion in human OASFs by reducing miR-5701 production through the PKC-α and JNK pathways. Our results also suggest that the development of IL-17, PKC, JNK, and VCAM-1 antagonists inhibit OA progression.

## 4. Materials and Methods

Antibodies against PKC-α, JNK, VCAM-1, CD11b, and β-actin were purchased from GeneTex (Hsinchu, Taiwan). Antibodies against p-PKCα and p-JNK were purchased from Cell Signaling Technology (Danvers, MA, USA). 2′,7′-Bis(2-carboxyethyl)-5(6)-carboxyfluorescein tetrakis(acetoxymethyl) ester (BCECF-AM) and GF109203X, Gö6976 and SP600125 inhibitors were obtained from Sigma-Aldrich (St. Louis, MO, USA).

### 4.1. Cell Culture

Synovial tissues freshly obtained from OA patients were washed with phosphate-buffered saline (PBS), minced thoroughly using a scalpel, and then subjected to 4 h of enzymatic digestion in serum-free DMEM medium with 2 mg/mL type I collagenase in an incubator at 37 °C, before removal of collagenase by centrifugation. OASFs were cultured in DMEM containing 10% fetal bovine serum (FBS), penicillin, and streptomycin (Invitrogen; Carlsbad, CA, USA) [25]. A total of 2 passages were performed of culture when the adherent cells approached 70% confluence, and experiments were performed using cells grown in vitro for 3–6 passages. 

THP-1 cells (human monocytes) were purchased from the Bioresource Collection and Research Center (Hsinchu, Taiwan) and maintained in RPMI-1640 medium supplemented with 10% FBS, 2 mM L-glutamine, 0.05 mM β-mercaptoethanol, 10 mM HEPES, 100 U/mL penicillin, and 100 μg/mL streptomycin at 37 °C in a humidified 5% CO_2_ atmosphere. THP-1 cells were kept at a minimum density of 3 × 10^5^ cells and were passaged when the density reached 8 × 10^5^ cells.

### 4.2. Monocyte Adhesion Analysis

Human OASFs (1 × 10^5^ cells) were cultured in 24-well culture plates and treated with IL-17 for 2 h, before changing the culture medium to serum-free conditions for 24 h. THP-1 cells were treated with BCECF-AM (10 μM; a pH-sensitive fluorescent dye used for determining living cells) at 37 °C for 1 h and subsequently washed twice with PBS by centrifugation. BCECF-AM-labeled THP-1 cells (2.5 × 10^5^ cells/mL) were added to a monolayer of OASFs at 37 °C for 1 h. Nonadherent THP-1 cells were cleaned off using PBS. Adherent THP-1 cells were quantified under fluorescence microscopy. We counted the number of cells in three random fields under each condition.

### 4.3. Human Clinical Samples

The collection of synovial samples from patients with OA and those with trauma/joint injuries (serving as normal, healthy controls) was approved by the Institutional Review Board of China Medical University Hospital. Informed written consent was obtained from all patients.

### 4.4. Real-Time Quantitative PCR Analysis of mRNA and miRNA

Total RNA was isolated from OASFs using TRIzol reagent (MDBio; Taipei, Taiwan). RNA (1 μg) was reverse-transcribed into cDNA with oligo-DT primer, according to the manufacturer’s procedure (Invitrogen; Carlsbad, CA, USA). qPCR was performed using SYBR Green with sequence-specific primers (Invitrogen; Carlsbad, CA, USA) (Supplementary Table S1). Levels of GAPDH or U6 snRNA expression served as the endogenous controls for normalization purposes. qPCR assays were performed with StepOnePlus (Applied Biosystems; Foster City, CA, USA) [11,40].

### 4.5. Western Blot

OASFs were treated with RIPA buffer. Isolated proteins were subjected to SDS-PAGE and transferred to polyvinylidene difluoride membranes (Merck; Darmstadt, Germany) [41,42]. The membranes were blocked with 5% nonfat milk and then treated with primary antibodies. The membranes were then washed and treated with secondary antibodies and then visualized using the ImageQuant™ LAS 4000 biomolecular imager [43,44,45].

### 4.6. Measurement of Data from the Gene Expression Omnibus (GEO) Database

Data on CD11b and VCAM-1 expression from normal, healthy control samples and OA samples were obtained from the GEO database, as according to our previous studies [46,47].

### 4.7. Immunohistochemistry (IHC)

Human synovial tissues were stained with anti-CD11b or VCAM-1 antibody and quantified according to the protocol described in our previous work [48,49]. The sum of the intensity and percentage scores was used as the final staining score [47].

### 4.8. Small Interfering RNA (siRNA) Transfection

ON-TARGETplus siRNAs of PKC (L-003523-00), JNK (L00351400), VCAM-1 (L-013351-00), and control (D0018101005) were purchased from Dharmacon Research (Lafayette, CO, USA). Transient transfection of siRNAs was carried out using DharmaFECT1 transfection reagent (T-2001-01). All siRNAs (100 nM) were formulated with DharmaFECT1 transfection reagent, according to the manufacturer’s instructions.

### 4.9. Statistical Analysis

All values are given as the mean ± standard deviation (S.D.). The Student’s *t*-test assessed between-group differences. A *p* value of < 0.05 was considered to be statistically significant.

## 5. Conclusions

**Figure 6:**
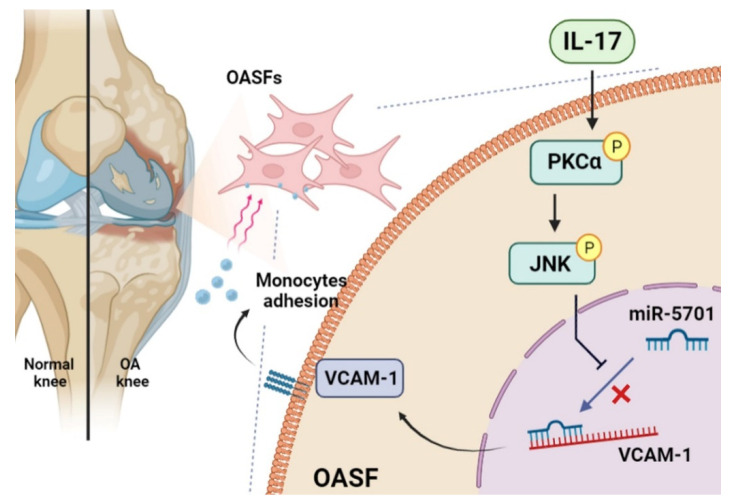
Schematic diagram summarizes the mechanisms by which IL-17 facilitates VCAM-1 production and monocyte adhesion in human OASFs. IL-17 promotes VCAM-1 synthesis and enhances monocyte adhesion in human OASFs by suppressing miR-5701 production in the PKC-α and JNK signaling cascades.

Our study indicates that IL-17 promotes VCAM-1 production in OASFs and facilitates monocyte adhesion by suppressing miR-5701 production via the PKC-α and JNK signaling cascades (Figure 6). We now have a better understanding about how IL-17-induced monocyte adhesion contributes to OA pathogenesis, which may help scientists design more effective therapy for OA.

## Data Availability

The original data to this present study are available from the corresponding authors.

## Supplementary Materials

The following supporting information can be downloaded at: https://www.mdpi.com/article/10.3390/ijms23126804/s1.
