# Supplementary material for: IL-17 Facilitates VCAM-1 Production and Monocyte Adhesion in Osteoarthritis Synovial Fibroblasts by Suppressing miR-5701 Synthesis
Source: Int J Mol Sci. 2022 Jun 18;23(12):6804. doi: 10.3390/ijms23126804 (PMC9224118; doi:10.3390/ijms23126804)
Supplement: Supplementary file 1 [file ijms-23-06804-s001.zip › ijms-1724693-supplementary.pdf]

International Journal of
Molecular Sciences

Supplementary data

Table S1. Primer sequences used in this study.

**Primers**	**Sequences (5’→3’)**
VCAM-1 (F)	TTTGACAGGCTGGAGATAGACT
VCAM-1 (R)	TCAATGTGTAATTTAGCTCGGCA
GAPDH (F)	ACCACAGTCCATGCCATCAC
GAPDH (R)	TCCACCACCCTGTTGCTGTA
hsa-let-7a-3p	CTATACAATCTACTGTCTTTC
hsa-let-7b-3p	CTATACAACCTACTGCCTTCCC
hsa-let-7f-1-3p	CTATACAATCTATTGCCTTCCC
hsa-let-7f-2-3p	CTATACAGTCTACTGTCTTTCC
hsa-miR-506-5p	TATTCAGGAAGGTGTTACTTAA
hsa-miR-561-5p	ATCAAGGATCTTAAACTTTGCC
hsa-miR-586	TATGCATTGTATTTTTAGGTCC
hsa-miR-1284	TCTATACAGACCCTGGCTTTTC
hsa-miR-3163	TATAAAATGAGGGCAGTAAGAC
hsa-miR-4282	TAAAATTTGCATCCAGGA
hsa-miR-4514	ACAGGCAGGATTGGGGAA
hsa-miR-4692	TCAGGCAGTGTGGGTATCAGAT
hsa-miR-4766-5p	TCTGAAAGAGCAGTTGGTGTT
hsa-miR-4775	TTAATTTTTGTTTCGGTCACT
hsa-miR-5580-5p	TGCTGGCTCATTTCATATGTGT
hsa-miR-5681a	AGAAAGGGTGGCAATACCTCTT
hsa-miR-5688	TAACAAACACCTGTAAAACAGC
hsa-miR-5701	TTATTGTCACGTTCTGATT
U6 (F)	GCTTCGGCAGCACATATACTAAAAT
U6 (R)	CGCTTCACGAATTTGCGTGTCAT

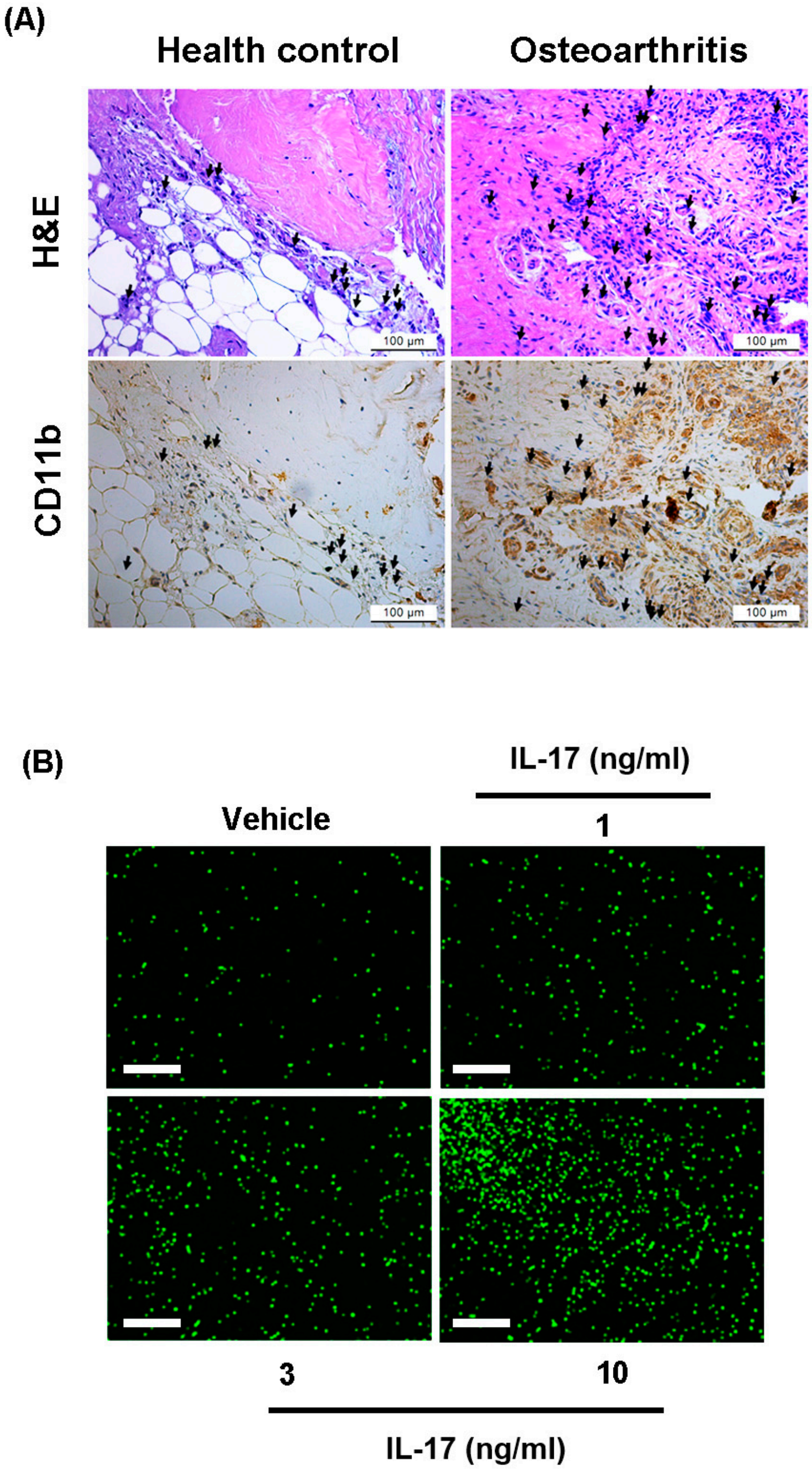

Figure S1. IL-17 promotes monocyte adhesion in OASFs. (A) IHC staining for CD11b in synovium samples from OA patients and healthy individuals. (B) OASFs were stimulated with vehicle or IL-17 (1–10 ng/mL) for 24 h. Monocytes (THP-1 cells) were then applied to the OASFs. Adherent THP-1 cells were photo-graphed and quantified under fluorescence microscopy. IHC size bar = 100 μm. Adhesion size bar = 320 μm.

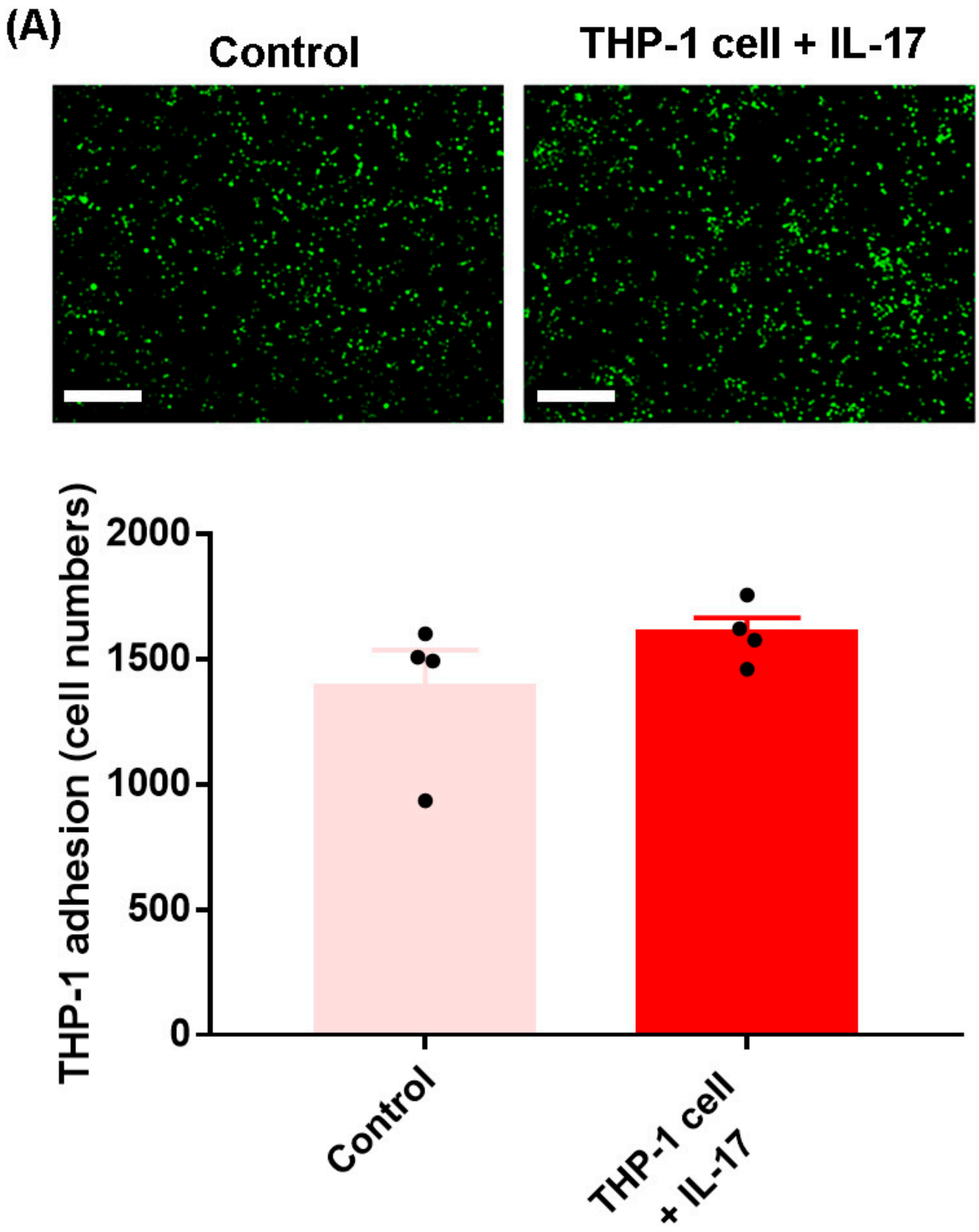

Figure S2. Stimulation of monocyte with IL-17 did not increase their adherence to OASFs. THP-1 cells were treated with IL-17 for 1 h then added to a monolayer of OASFs. Adherent THP-1 cells were photographed and quantified under fluorescence microscopy. Adhesion size bar = 320 μm.

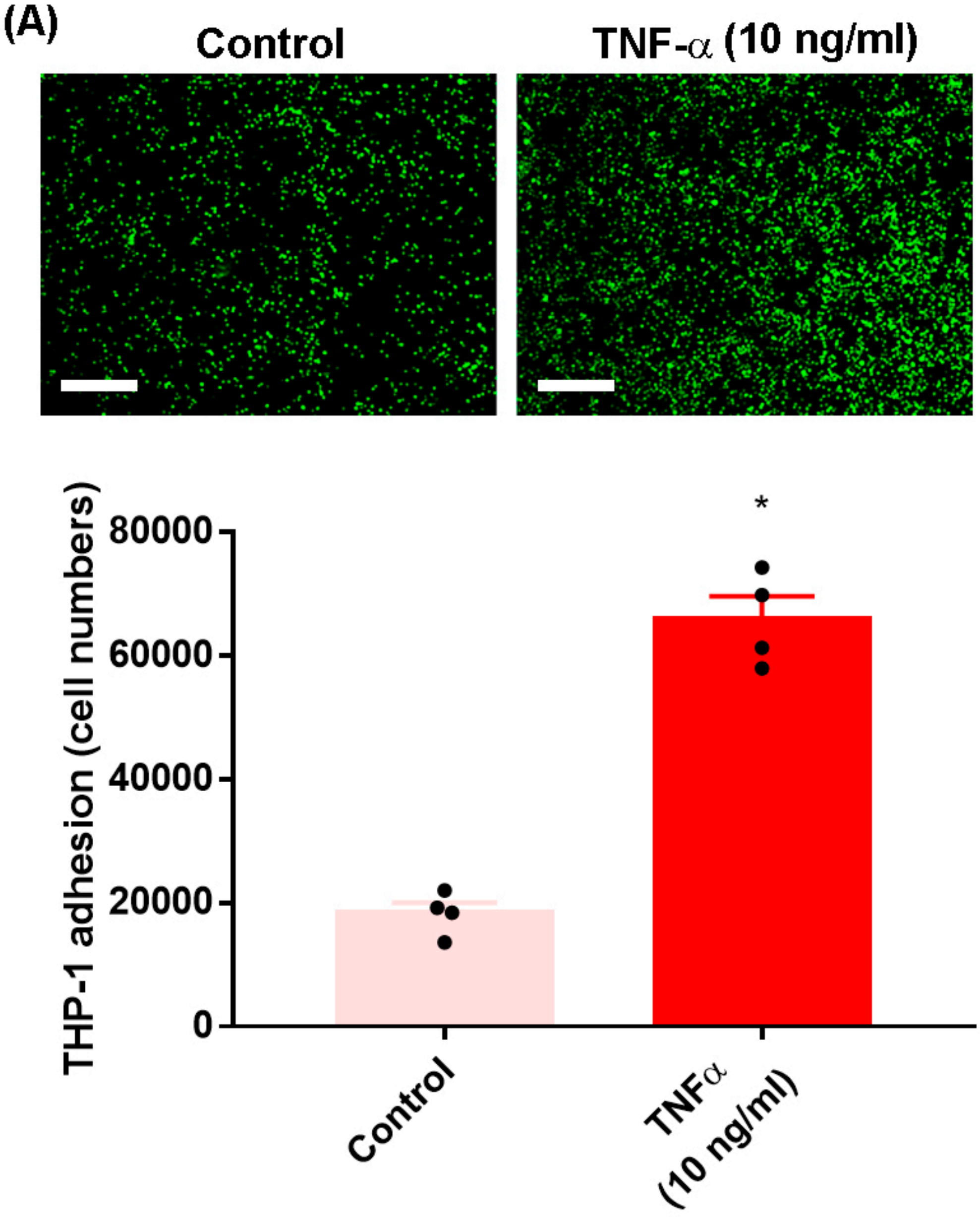

Figure S3. Stimulation of OASFs with TNF-*α* (10 ng/ml) promote monocyte adherence. OASFs were stimulated with TNF-α. Adherent THP-1 cells were photographed and quantified under fluorescence microscopy. Adhesion size bar = 320 μm. * $p < 0.05$ compared with the control group.

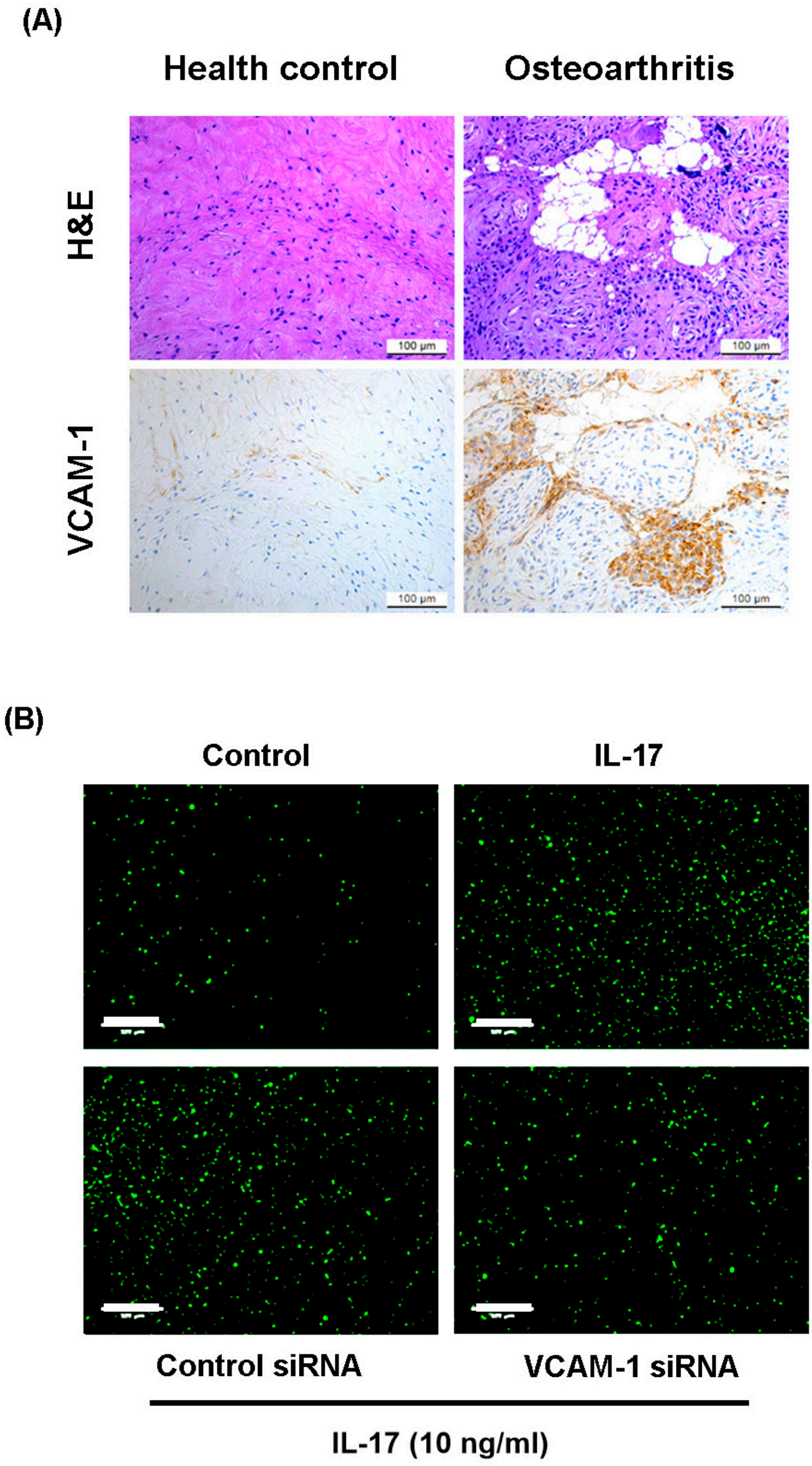

Figure S4. IL-17 promotes monocyte adhesion in OASFs through upregulating VCAM-1 production. (A) IHC staining for VCAM-1 in synovium samples from OA patients and healthy individuals. (B) OASFs were transfected with a VCAM-1 siRNA for 24 h and then treated with IL-17 (10 ng/mL). Adherent THP-1 cells were photographed and quantified under fluorescence microscopy. IHC size bar = 100 μm. Adhesion size bar = 320 μm.

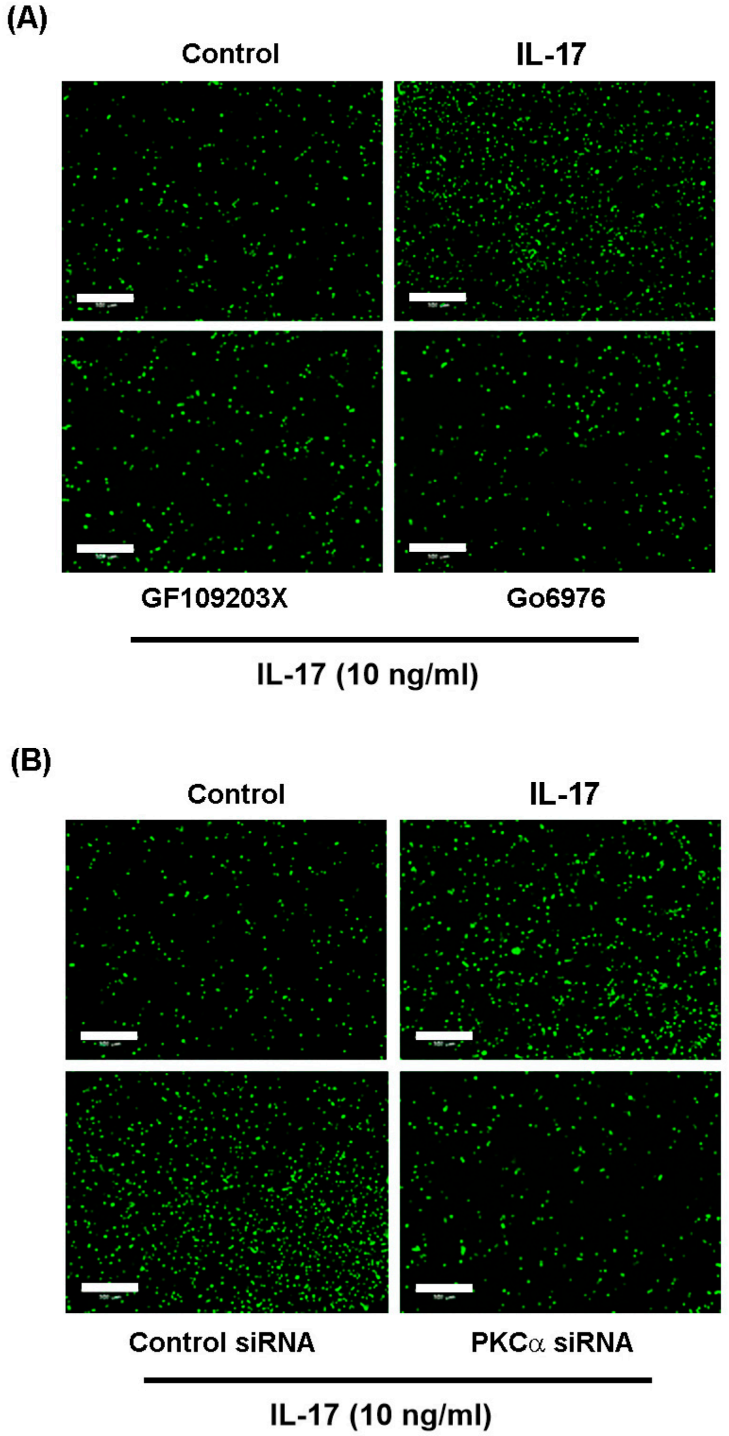

Figure S5. The PKC-α pathway mediates IL-17-induced promotion of VCAM-1 synthesis and monocyte adhesion in OASFs. (A&B) OASFs were stimulated with PKC inhibitors (GF109203X, 10 nM and Gö6976, 10 nM) for 30 min or transfected with a PKC-α siRNA for 24 h and then treated with or without IL-17 (10 ng/mL) for 24 h. Adherent THP-1 cells were photographed and quantified under fluorescence microscopy. Adhesion size bar = 320 μm.

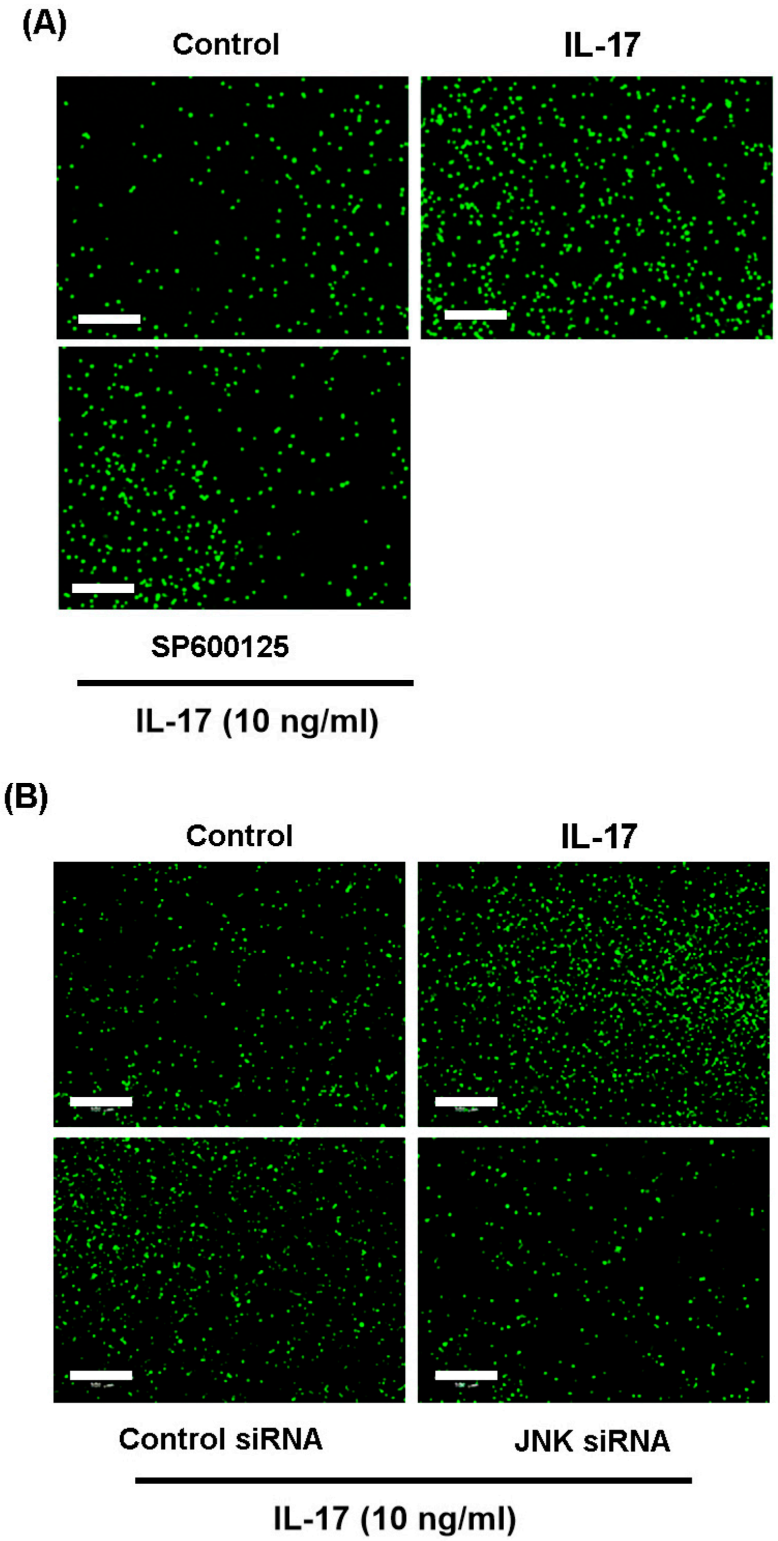

Figure S6. The JNK pathway mediates IL-17-induced promotion of VCAM-1 synthesis and monocyte adhesion in OASFs. (A&B) OASFs were stimulated with a JNK inhibitor (SP600125, 10 nM) for 30 min or transfected with a JNK siRNA for 24 h and then treated with IL-17 (10 ng/mL) for 24 h, the adherent THP-1 cells were photographed and quantified under fluorescence microscopy. Adhesion size bar = 320 μm.

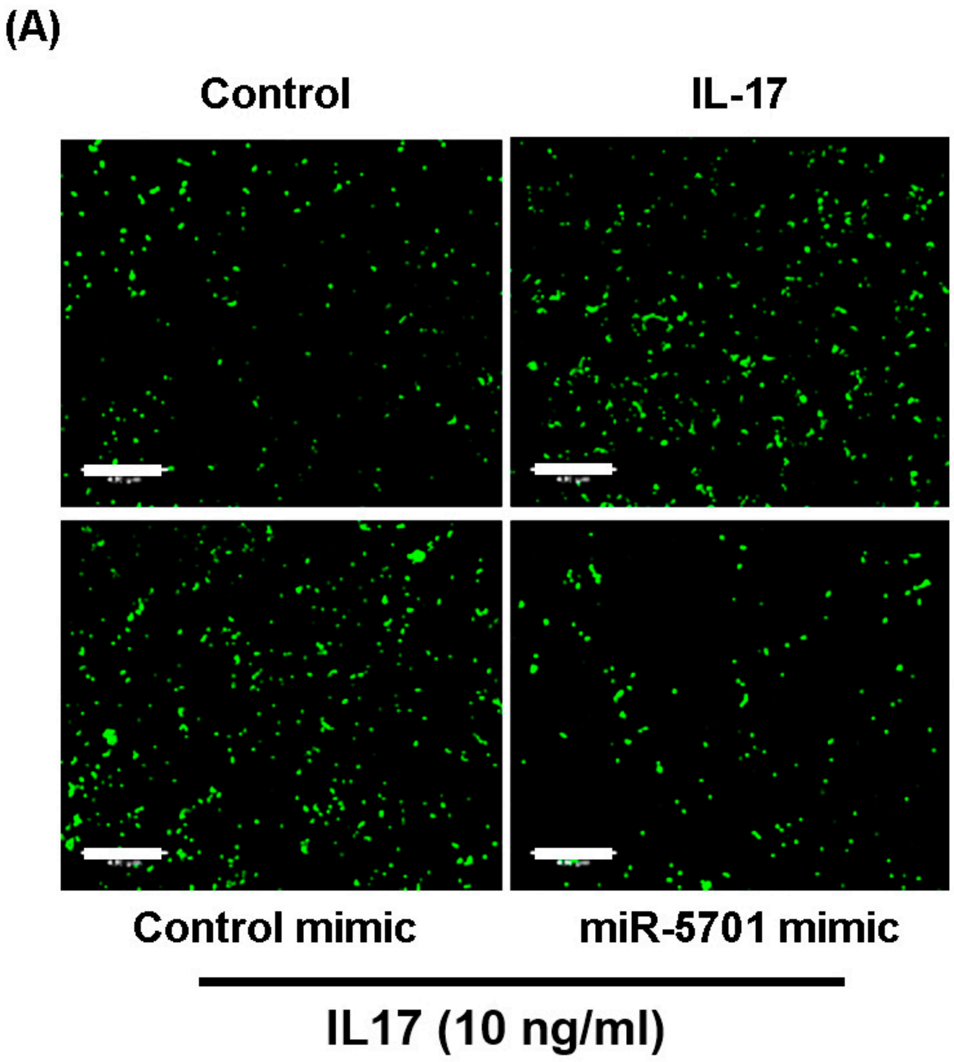

Figure S7. IL-17 enhances VCAM-1 production by inhibiting miR-5701 expression. (A) OASFs were transfected with miR-5701 mimic and then treated with IL-17 (10 ng/mL). Adherent THP-1 cells were photographed and quantified under fluorescence microscopy. Adhesion size bar = 320 μm.
